# Gene Expression Signatures of Synovial Fluid Multipotent Stromal Cells in Advanced Knee Osteoarthritis and Following Knee Joint Distraction

**DOI:** 10.3389/fbioe.2020.579751

**Published:** 2020-10-14

**Authors:** Clara Sanjurjo-Rodriguez, Ala Altaie, Simon Mastbergen, Thomas Baboolal, Tim Welting, Floris Lafeber, Hemant Pandit, Dennis McGonagle, Elena Jones

**Affiliations:** ^1^Leeds Institute of Rheumatic and Musculoskeletal Medicine, University of Leeds, Leeds, United Kingdom; ^2^Physiotherapy, Medicine and Biomedical Sciences department, CIBER-BBN, Institute of Biomedical Research of A Coruña (INIBIC)-Centre of Advanced Scientific Researches (CICA), University of A Coruña, A Coruña, Spain; ^3^University Medical Center Utrecht, Rheumatology & Clinical Immunology, Regenerative Medicine Center Utrecht, Utrecht University, Utrecht, Netherlands; ^4^Laboratory for Experimental Orthopedics, Department of Orthopedic Surgery, Maastricht University Medical Center, Maastricht, Netherlands; ^5^NIHR Leeds Musculoskeletal Biomedical Research Centre, Leeds, United Kingdom

**Keywords:** multipotent stromal cells, synovial fluid, osteoarthritis, knee joint distraction, subchondral bone, chondrocytes

## Abstract

Osteoarthritis (OA) is the most common musculoskeletal disorder. Although joint replacement remains the standard of care for knee OA patients, knee joint distraction (KJD), which works by temporarily off-loading the joint for 6–8 weeks, is becoming a novel joint-sparing alternative for younger OA sufferers. The biological mechanisms behind KJD structural improvements remain poorly understood but likely involve joint-resident regenerative cells including multipotent stromal cells (MSCs). In this study, we hypothesized that KJD leads to beneficial cartilage-anabolic and anti-catabolic changes in joint-resident MSCs and investigated gene expression profiles of synovial fluid (SF) MSCs following KJD as compared with baseline. To obtain further insights into the effects of local biomechanics on MSCs present in late OA joints, SF MSC gene expression was studied in a separate OA arthroplasty cohort and compared with subchondral bone (SB) MSCs from medial (more loaded) and lateral (less loaded) femoral condyles from the same joints. In OA arthroplasty cohort (*n* = 12 patients), SF MSCs expressed lower levels of ossification- and hypotrophy-related genes [bone sialoprotein (IBSP), parathyroid hormone 1 receptor (PTH1R), and runt-related transcription factor 2 (RUNX2)] than did SB MSCs. Interestingly, SF MSCs expressed 5- to 50-fold higher levels of transcripts for classical extracellular matrix turnover molecules matrix metalloproteinase 1 (MMP1), a disintegrin and metalloproteinase with thrombospondin motifs 5 (ADAMTS5), and tissue inhibitor of metalloproteinase-3 (TIMP3), all (*p* < 0.05) potentially indicating greater cartilage remodeling ability of OA SF MSCs, compared with SB MSCs. In KJD cohort (*n* = 9 patients), joint off-loading resulted in sustained, significant increase in SF MSC colonies’ sizes and densities and a notable transcript upregulation of key cartilage core protein aggrecan (ACAN) (weeks 3 and 6), as well as reduction in pro-inflammatory C–C motif chemokine ligand 2 (CCL2) expression (weeks 3 and 6). Additionally, early KJD changes (week 3) were marked by significant increases in MSC chondrogenic commitment markers gremlin 1 (GREM1) and growth differentiation factor 5 (GDF5). In combination, our results reveal distinct transcriptomes on joint-resident MSCs from different biomechanical environments and show that 6-week joint off-loading leads to transcriptional changes in SF MSCs that may be beneficial for cartilage regeneration. Biomechanical factors should be certainly considered in the development of novel MSC-based therapies for OA.

## Introduction

Multipotent stromal cells (MSCs), originally termed as mesenchymal stem cells, are present in different joint structures including the synovial membrane, synovial fluid (SF), infrapatellar fat pad, ligaments/tendons, and the subchondral bone (SB) (Fellows et al., 2016; McGonagle et al., 2017). Several studies have also described the presence of MSC-like chondrogenic progenitor cells (CPCs) in the articular cartilage (Schminke and Miosge, 2014; Jayasuriya et al., 2018). SF MSCs are a particularly intriguing class of joint-resident MSCs, as unlike other MSCs, they are not attached to other cells or surfaces but instead are suspended in a viscous hyaluronan-rich SF (Jones et al., 2004; Baboolal et al., 2016; Fang et al., 2020). In healthy individuals, they are believed to be shed from the synovial intimal layer or superficial cartilage, as a result of mechanical attrition during joint movement (Jones et al., 2008; Morito et al., 2008; Lee et al., 2012; Matsukura et al., 2014). Healthy SF MSCs are highly proliferative and consistently chondrogenic (Jones et al., 2008; Katagiri et al., 2017). Combined with their ease of harvesting, despite their limited numbers, they are considered as an attractive MSC source for repairing focal osteochondral defects and potentially, joint regeneration in osteoarthritis (OA), acting via direct differentiation (McGonagle et al., 2017; Piñeiro-Ramil et al., 2017) as well as paracrine mechanisms (Garcia et al., 2016).

On the other hand, MSCs present in the SB are accessible through subchondral plate perforation and are believed to be responsible for limited cartilage regeneration following microfracture treatment of focal cartilage lesions (Dwivedi et al., 2018). In our previous work, we investigated SB MSCs in advanced knee OA and compared MSCs from the medial, commonly weight-bearing compartment, with the lateral side of the joint (Sanjurjo-Rodriguez et al., 2019). This work used collagenase-assisted release of MSCs from subchondral trabecular bone surfaces and marrow-filled bone cavities and demonstrated large MSC numbers in both lateral and medial femoral condyles. However, in more damaged, medial condyles, SB MSCs expressed higher levels of ossification-related genes compared with less-damaged lateral condyles, indicating their preference for sclerotic bone formation rather than cartilage restoration and highlighting key contribution on joint biomechanics in MSC pathophysiology in OA (Sanjurjo-Rodriguez et al., 2019). These findings are in line with previous OA animal model studies as well as our previous work on human hip OA that showed an aberrant osteogenesis of MSCs in OA SB (Zhen et al., 2013; Ilas et al., 2019, 2020).

Multipotent stromal cells numbers are increased in OA SF (Jones et al., 2008), in direct correlation with disease severity (Jones et al., 2008; Sekiya et al., 2012; Harris et al., 2013; Kim et al., 2015). This has been assumed to represent joint’s repair attempt, but the source of these additional MSCs and their cartilage-supportive properties remains unclear. We and others have previously proposed that additional MSCs are shed from the synovium and accumulate inside the fluid (Jones et al., 2008; Katagiri et al., 2017), as a result of their enhanced proliferation and possibly, reduced attachment to joint structures (Jones et al., 2008; Harris et al., 2013). Another possibility is that at least a subset of these OA SF MSCs is liberated from the damaged articular cartilage, which is the prime “victim” of tissue destruction in OA (Katagiri et al., 2017). The presence of “migratory CPCs” in deep OA articular cartilage has been previously described (Koelling et al., 2009), and these CPCs may in principle enter the joint space via cracks and fissures in degenerated cartilage. In the very advanced OA stages, additional MSCs may also originate from the progressively exposed SB (Koelling et al., 2009; Iijima et al., 2016).

Knee joint distraction (KJD) is an emerging treatment for OA (Takahashi et al., 2019; Jansen et al., 2020), which is associated with impressive structural and clinical outcomes (van der Woude et al., 2017a; Jansen et al., 2018). It works by simply taking the load off the joint by surgically pulling the joint apart using an external fixation frame, which is placed on both sides of the joint, allowing distraction for a few millimeters for up to 6–8 weeks (van der Woude et al., 2017a). It is considered a good treatment option for the younger OA subjects in whom replacement might be premature and more likely to result in early failure (Schreurs and Hannink, 2017). Patients with established OA demonstrate improved knee symptoms for 5 to 9 years after the KJD (van der Woude et al., 2017b; Jansen et al., 2018). The 6-week period of the off-load leads to apparent cartilage regeneration, with increase in joint space width (JSW) on X-ray and increased articular cartilage thickness on magnetic resonance imaging (MRI) (Wiegant et al., 2013; Jansen et al., 2018, 2019). First-year minimum JSW on radiographs and cartilage thickness increase on MRI are predictive of the 9-year results (Jansen et al., 2018). As such, the initial cartilage repair activity appears to be important for long-term clinical success. This suggests that, by temporarily off-loading the joint, KJD might trigger the intrinsic cartilaginous repair, which may be facilitated by SF MSCs. Indeed, in the canine groove model of OA, we showed that MSCs injected in the SF, following KJD, were capable of integrating into cartilage injury sites (Baboolal et al., 2016). Furthermore, a recent study provided evidence for anabolic molecular responses in SF following KJD, which may act on SF MSCs and represent potential pathways for cartilage regeneration (Watt et al., 2020).

Based on the fact that SF MSCs and SB MSCs may be present endogenously at sites of cartilage damage in OA, and that denuded bone may serve as a potential source of SF MSCs in advanced OA, we compared their gene expression signatures and assessed the MSC specificity of differentially expressed transcripts by comparing them with cultured chondrocytes from the same joints. We hypothesized that in comparison with SB MSCs, SF MSCs that are highly chondrogenic in healthy individuals may be a better MSC population for endogenous manipulation and cartilage regeneration in OA and that following KJD, their gene expression signature may change in favor of cartilage regeneration. The aims of this study were therefore to investigate gene expression signatures of SF MSCs in advanced knee OA, in comparison with SB MSCs from the same joints, and to investigate gene expression changes in SF MSCs following KJD.

## Materials and Methods

### Patients and Samples

This study was performed in compliance with the Declaration of Helsinki of ethical principles for medical research involving human subjects. Ethical approval was obtained from the Yorkshire & The Humber–South Yorkshire Research Ethics Committee (14/YH/0087). For KJD study, ethical approval was obtained from the medical ethical review committee of the University Medical Center Utrecht (#15-160/D; NL51539.041.15).

Twelve patients who underwent total knee arthroplasty (median age 72 years, range 61–83; seven women and five men) were recruited after informed written consent was given, and both lateral and medial femoral condyle samples were transferred to the laboratory (Supplementary Figure 1A). From six patients (median age 80 years, range 64–83; three men and three women), donor-matched SFs were also collected using a syringe, after opening the joint cavity for the arthroplasty.

Patients with established symptomatic radiographic knee OA undergoing KJD gave written informed consent to participate (*n* = 9, median 51 age years, range 35–60; two men and seven women). SF was sampled at baseline (before distraction; pre), during (3 weeks; during), and at endpoint of distraction (6 weeks; post).

### Tissue Processing for Multipotent Stromal Cell and Chondrocyte Isolation

#### Multipotent Stromal Cell Isolation and Expansion

For isolation of MSCs from SF, the fluid was diluted 1:4 in phosphate-buffered saline (PBS) and centrifuged 500 × *g* for 10 min, and pelleted cells were seeded in T25 flasks with StemMACS MSC Expansion Media (Miltenyi Biotec, Germany) supplemented with 1% penicillin/streptomycin (P/S; Thermo Fisher Scientific).

For SB MSC isolation, following cartilage removal using a scalpel, bone from the separate medial and lateral condyles was mechanically minced into small fragments with a rongeur and digested with 3,000 units of collagenase/g of tissue (Worthington Biochem Corp., United States) for 4 h (Supplementary Figure 1A), as previously described (Sanjurjo-Rodriguez et al., 2019). The supernatant was filtered through 22-μm cell strainer (Corning Inc., United States) before centrifugation at 450 × *g* for 10 min to pellet the extracted cells. After being counted, the cells were seeded for culture expansion at the seeding density of 4.0 × 10^4^ cells/cm^2^ in flasks containing StemMACS MSC Expansion Media supplemented with 1% P/S.

Media were changed twice a week, and cells were split when 80% confluence was reached for both types of MSCs. Part of passage 1 SB and SF MSCs were used for gene expression study, and passage 3–5 SF cells were used for MSC characterization. Accrued population doublings (PDs) and PD rates were calculated as previously described (Churchman et al., 2012).

#### Chondrocyte Isolation and Expansion

All articular cartilage was harvested from the lateral and medial condyle surfaces using a scalpel, and chondrocytes were isolated as described before and kept separate (Sanjurjo-Rodriguez et al., 2019). We were interested to see whether the nature of cells (MSCs versus chondrocytes) or their loading environment (medial versus lateral) had a stronger effect on their gene expression. Briefly, cartilage was minced using a scalpel and digested overnight with 3,000 units of collagenase/g of tissue, and the homogenate was filtered with 22-μm cell strainer. The supernatant containing cells was centrifuged at 450 × *g* for 10 min, cell pellet was digested 5 min with 1 × trypsin Thermo Fisher Scientific, United States), and the cells were seeded into flasks (Corning Inc.) in Dulbecco’s modified Eagle’s medium (DMEM; Thermo Fisher Scientific) with 10% fetal bovine serum (FBS; BioSera, France) and 1% P/S at the seeding density of 1.2 × 10^4^ cells/cm^2^. Media were changed every 3–4 days, and subculture was performed when cells reached 80% confluence. Passage 1-cultured chondrocytes were used for gene expression analysis as controls for SB and SF MSCs.

### Colony-Forming Unit-Fibroblast Assay

Synovial fluid samples were diluted 1:4 in sterile PBS and thoroughly mixed before centrifuging at 500 × *g* for 5 min. Cells were resuspended in 1 ml of warmed StemMACS MSC Expansion media (Miltenyi Biotec, Germany) supplemented with P/S. Next, cells were diluted with 9 ml of StemMACS media to a final volume of 10 ml. Samples were plated in duplicate on colony-forming unit-fibroblast (CFU-F) dishes (60 mm) by adding 3 ml of the sample and an additional 2 ml of StemMACS to final volume of 5 ml per dish. The remaining sample (∼4 ml) was used for MSC expansion and mRNA analysis. Subsequently, the dishes were incubated at 37°C and 5% CO_2_ in a humidified incubator. After 2–3 days, the media were removed and replaced with fresh StemMACS media. Thereafter, half of the media (2.5 ml) were refreshed twice weekly. After 14 days, all media were removed, and cells fixed with 3.7% formalin (buffered with PBS) for 30 min at room temperature. Formalin was removed, and the dishes were gently washed with water.

The fixed dishes were stained with methylene blue for CFU-F counting and were scanned for CFU-F area and integrated density (ID) analysis, as previously described (Ganguly et al., 2019; Sanjurjo-Rodriguez et al., 2019). Briefly, scanned images were converted to 8-bit format, calibrated, and analyzed using ImageJ software. Colony area and ID were measured independently in all colonies after thresholding.

### Synovial Fluid Multipotent Stromal Cell Characterization

Characterization of SF MSCs was performed following the minimal criteria from International Society for Cellular Therapy (ISCT) (Dominici et al., 2006).

#### Surface Marker Expression

Synovial fluid MSCs from three randomly selected donors were characterized by flow cytometry according to the ISCT criteria. The antibodies used were CD90-FITC (AbD Serotec, Kidlington, UK), CD73-PE and CD105-PE (both from BD Biosciences, Wokingham, UK) (MSC positive markers) and CD19-FITC, CD14-PE, CD34-PE, CD45-V450, and HLA-DR-fluorescein isothiocyanate (FITC) (hematopoietic lineage markers) (all from BD Biosciences). A minimum of 10,000 cell events per tube was acquired using Attune flow cytometer (Applied Biosystems). Data were analyzed using FlowJo (BD), and the results are expressed as percentage of positive cells.

#### Tri-Lineage Differentiation and Motility Assay

Tri-lineage differentiation was performed on the same cultures, as described before (Sanjurjo-Rodriguez et al., 2019). Briefly, SF MSCs were seeded in 24-well plates (Corning) for adipogenic and osteogenic differentiation. Cells were cultured in adipogenic media containing DMEM supplemented with 12.5% FBS, 12.5% horse serum, 0.5 mM of isobutylmethylxanthine, 60 μM of indomethacin, and 0.5 mM of hydrocortisone (all from Sigma) for 21 days; and adipogenesis was assessed by Oil Red O staining. Osteogenesis was assessed by Alizarin Red staining after 21-day culture in osteogenic media containing DMEM supplemented with 10% FBS, 100 nM of dexamethasone, 0.05 mM of ascorbic acid, and 100 mM of β-glycerosphosphate (all from Sigma).

Chondrogenic differentiation was performed with ChondroDiff media (Miltenyi Biotec) in a three-dimensional (3D) pellet culture for 21 days. Toluidine blue staining was performed in 5-μm paraffin-sectioned pellets to evaluate glycosaminoglycan (GAG) content.

*In vitro* motility of SB and SF MSCs was assessed using a scratch assay as described before (Sanjurjo-Rodriguez et al., 2019). Briefly, MSCs were grown to confluence in a 6-well plate in StemMACS MSC Expansion Media. The media were removed, and the cell monolayer was scratched with a sterile 200-μl pipette tip. The well was washed with PBS, and new fresh expansion media were added. Images were taken along the open scratch at 0 and 24 h, and measurements were calculated using the ImageJ software. The percentage of uncovered area (wound open) was normalized to time 0 and compared between SF MSCs and SB MSCs.

### Gene Expression

RNA was isolated from MSCs and chondrocyte cultures, using the total RNA Purification kit (Norgen Biotek Corp., Canada). Gene expression was performed for 95 genes of interest as described in our previous work (Sanjurjo-Rodriguez et al., 2019), using standard TaqMan Assays (Thermo Fisher Scientific) and the 48.48 IFC (Integrated Fluidic Circuit) with the recommended reagents (Fluidigm Corporation, United States), following manufacturer’s recommendations. Hypoxanthine phosphoribosyltransferase 1 (HPRT1) was used as a housekeeping gene. Reverse transcription was performed, followed by 14 pre-amplification cycles using a mixture of 96 TaqMan gene expression assays (Supplementary Table 1). The Dynamic 48.48 IFC sample compartment was loaded with the diluted pre-amplified cDNAs mixed with sample loading buffer (Fluidigm Corporation) and TaqMan Universal PCR Master mix (Applied Biosystems, United States). The IFC assay compartment was loaded with the 96 TaqMan assays mixed with assay loading buffer (Fluidigm Corporation). The IFC was then run on the Biomark Real Time PCR System using a GE 48 × 48 Standard v1 PCR thermal protocol, and data were analyzed using BioMark Gene Expression Data software and normalized to the housekeeping gene. Genes differentially expressed between chondrocytes, SB, and SF MSCs were further scrutinized for hierarchical clustering analysis using Cluster 3.0 software and Java TreeView (Churchman et al., 2012), including only samples that expressed ≥60% of all genes and genes expressed in ≥80% of the samples.

### Statistical Analysis

Results were analyzed using Kruskal–Wallis and Dunn’s multiple comparison tests for unpaired data and Wilcoxon signed rank tests for donor-matched data. The statistical analysis was performed using Prism software (version 7.0 a; GraphPad). The difference between the groups was considered as statistically significant only if the *p* value <0.05.

## Results

### Osteoarthritis Synovial Fluid Multipotent Stromal Cell Characterization

**FIGURE 1:**
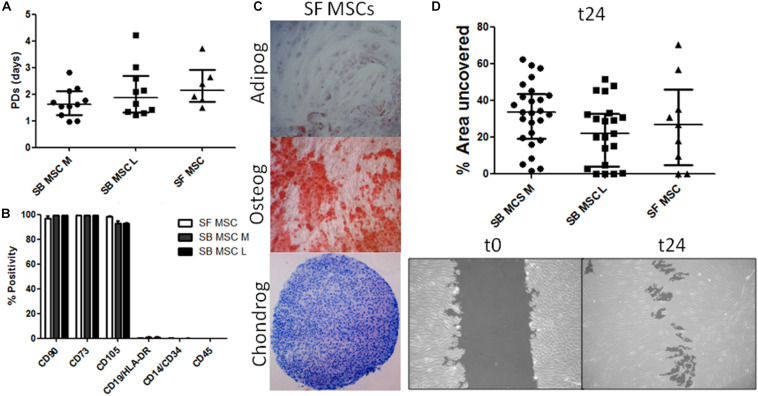
Phenotypical and functional analyses of multipotent stromal cells (MSCs). **(A)** Comparison of growth rates of medial and lateral subchondral bone (SB) MSCs (SB MSC M and SB MSC L, respectively) and synovial fluid (SF) MSCs measured as population doubling (PD) times (in days). Dots represent individual donors; error bars represent interquartile range (IQR). **(B)** Phenotypic profile of culture-expanded SF and medial and lateral SB MSCs indicating no differences in the expression of standard MSC markers. Horizontal bars show means and error bars represent standard error (*n* = 3 donors). **(C)** Example images of SF MSC adipogenesis assay (Adipog) stained with Oil Red O after 14 days of adipogenic induction (top panel, original magnification ×100), osteogenesis (Osteog) showing positive alizarin red staining on day 14 post osteogenic induction (medium panel, original magnification ×100), and toluidine blue staining for glycosaminoglycan (GAG) deposition (purple) of chondrogenic (Chondrog) pellet cultures on day 21 post induction (bottom panel, original magnification ×40). **(D)** Analysis of SF MSC motility showing no significant differences between MSCs. Top panel: graph representing the median percentage of wound uncovered by migrating cells after 24 h (relative to the corresponding 0-h area); data represent individual measurements from nine SB MSC donors and three SF MSC donors; error bars represent IQR. Bottom panels: example images showing uncovered area measured at time 0 and at 24 h (t24).

Synovial fluid MSC cultures were established according to standard methods (Jones et al., 2004). SB MSC cultures were derived and characterized from both medial and lateral femoral condyles, as previously described (Sanjurjo-Rodriguez et al., 2019). There was no significant difference in the growth rates or surface phenotypes of SF MSC cultures and both types of SB MSCs (Figures 1A,B). Consistent with their MSC nature, SF cultures were tri-potential (Figure 1C). In motility assays, SF MSCs moved slightly faster than medial SB MSCs but were slower that lateral SB MSCs, and the differences failed to reach statistical significance (Figure 1D).

### Gene Expression Differences Between Synovial Fluid Multipotent Stromal Cells, Subchondral Bone Multipotent Stromal Cells, and Chondrocytes

**FIGURE 2:**
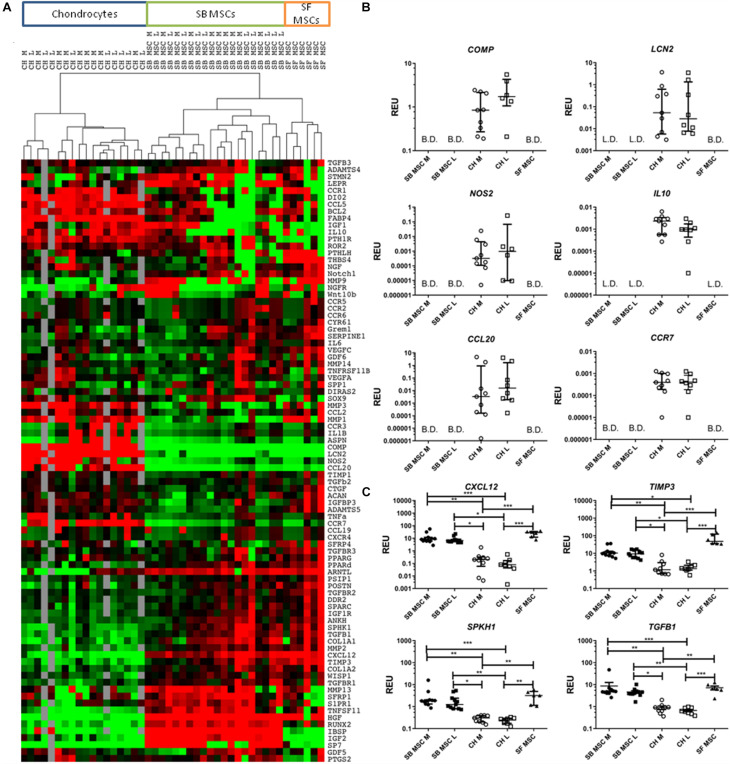
Gene expression analysis of culture-expanded medial and lateral subchondral bone (SB) multipotent stromal cells (MSCs) (SB MSC M and SB MSC L, respectively), synovial fluid (SF) MSCs, and medial and lateral chondrocytes (CH M and CH L, respectively). **(A)** Cluster analysis between CH, SB MSCs, and SF MSCs illustrating clear clustering of MSCs away from chondrocytes. Data were normalized to the housekeeping gene HPRT and log2 transformed. Data filtering was performed according to standard methods previously described (Churchman et al., 2012). Scores were assigned as: black = 1, red > 1, green < 1; gray = missing data (below detection). **(B)** Differentially expressed genes with a significant higher expression in chondrocytes than in MSCs. L.D. indicates transcripts which were rarely expressed (in < 50% samples), and B.D. indicates transcripts below detection. **(C)** Differentially expressed genes between chondrocytes and MSCs, which were statistically significantly higher expressed in MSCs than in chondrocytes. *p < 0.05; **p < 0.01; ***p < 0.0001. Horizontal bars show medians and error bars represent interquartile range (IQR). REU, relative expression units [relative to housekeeping hypoxanthine phosphoribosyltransferase (HPRT)].

Global gene expression differences between all culture types were first investigated using cluster analysis and revealed a clear clustering of SF MSCs and SB MSCs away from cultured chondrocytes from the same joints (Figure 2A). Also, within MSCs, a clear sub-cluster of SF MSCs was evident. Statistical analysis was next performed to identify differentially expressed genes between all MSCs and all chondrocytes (Figures 2B,C). The most differentially expressed genes highly expressed in chondrocytes were as follows: cartilage oligomeric matrix protein (COMP), lipocalin 2 (LCN2), nitric oxide synthase 2 (NOS2), interleukin 10 (IL10), C–C motif chemokine ligand 20 (CCL20), also known as macrophage inflammatory protein-3, and C–C motif chemokine receptor 7 (CCR7). All of these genes were expressed in cultured chondrocytes and low or below detection in both SF and SB MSCs (Figure 2B).

In contrast, four genes were significantly higher expressed in both SB and SF MSCs, compared with cultured chondrocytes (Figure 2C). These genes were C–X–C motif chemokine 12 (CXCL12, 58-fold higher in SB MSCs and 234-fold higher in SF MSCs), tissue inhibitor of metalloproteinase-3 (TIMP3; 7.7-fold higher in SB MSCs and 36.7-fold higher in SF MSCs), sphingosine kinase 1 (SPHK1; 6.5-fold higher in SB MSCs and 11.9-fold higher in SF MSCs), and transforming growth factor beta 1 (TGFB1; 6.9-fold higher in SB MSCs and 9.6-fold higher in SF MSCs). None of these four genes was significantly different between SB and SF MSCs. These data indicated that as expected, SF MSC gene expression signature was more similar to SB MSCs than cultured chondrocytes.

### Gene Expression Comparison Between Synovial Fluid Multipotent Stromal Cells and Medial and Lateral Subchondral Bone Multipotent Stromal Cells

Synovial fluid MSC gene expression signature was next compared with medial and lateral SB MSCs. When gene expression profiles of SF MSCs were compared with those of medial SB MSCs, 82% of the measured genes showed similar levels of expression (Supplementary Table 1), 7% genes showed significantly >2-fold higher expression in medial SB MSCs, and 11% of genes were significantly >2-fold higher expressed in SF MSCs (Table 1). Similarly, when gene expression profiles of SF MSCs were compared with lateral SB MSCs, 69% of genes showed similar levels of expression (Supplementary Table 1), 5% genes showed significantly >2-fold higher expression in lateral SB MSCs, and 26% of genes were significantly >2-fold higher expressed in SF MSCs (Table 1). Interestingly, genes previously described as highly specific for synovial-origin MSCs, such as SFRP4 (Sekiya et al., 2012; Baboolal et al., 2014) and growth differentiation factor 5 (GDF5) (Kania et al., 2020), were not expressed at the higher levels in OA SF MSCs (Table 1).

**TABLE 1:** Gene expression differences between SF and either lateral or medial SB MSCs.

Lower expression in SF than SB MSCs					
Gene symbol	Gene name	Medial SB MSCs > SF		Lateral SB MSCs > SF	
		Fold difference	*p*-value	Fold difference	*p*-value
SP7	Sp7 transcription factor	SF B.D.	N/A	SF B.D.	N/A
IBSP	Integrin binding sialoprotein	SF L.D.	N/A	SF L.D.	N/A
LEPR	Leptin receptor	SF L.D.	N/A	SF L.D.	N/A
PTH1R	Parathyroid hormone 1 receptor	SF L.D.	N/A	SF L.D.	N/A
RUNX2	Runt-related transcription factor 2	3.94	<0.05	6.38	<0.01
IGF2	Insulin-like growth factor 2	3.13	<0.05	4.83	<0.01
PTGS2	Prostaglandin-endoperoxide synthase 2	3.79	<0.05	4.03	NS

**Higher expression in SF than SB MSCs**					
**Gene symbol**	**Gene name**	**SF > medial SB MSCs**		**SF > lateral SB MSCs**	
		**Fold difference**	***p*-value**	**Fold difference**	***p*-value**

CCL5	C–C motif chemokine ligand 5	Med L.D.	N/A	Lat L.D.	N/A
PTHLH	Parathyroid hormone-like hormone	6.35	<0.05	Lat L.D.	N/A
MMP9	Matrix metalloproteinase 9	4.28	NS	Lat L.D.	N/A
STMN2	Stathmin 2	11.57	NS	Lat L.D.	N/A
ACAN	Aggrecan	1.93	<0.05	2.21	NS
THBS4	Thrombospondin 4	57.12	<0.05	78.52	<0.01
MMP1	Matrix metalloproteinase 1	36.95	<0.05	52.90	<0.01
CCR1	C–C motif chemokine receptor 1	8.08	<0.05	10.52	<0.05
ADAMTS5	A disintegrin and metalloproteinase with thrombospondin motifs 5	10.32	<0.01	8.47	<0.01
VEGFC	Vascular endothelial growth factor C	5.38	<0.01	5.46	<0.01
TIMP3	Tissue inhibitor of metalloproteinase-3	4.60	<0.01	5.20	<0.01
POSTN	Periostin	2.06	<0.05	2.59	<0.05
TGFBR3	Transforming growth factor beta receptor 3	2.59	<0.05	3.43	<0.01
TGFBR2	Transforming growth factor beta receptor 2	2.55	NS	4.87	<0.001
DDR2	Discoidin domain receptor tyrosine Kinase 2	2.31	NS	2.33	<0.01
IGFBP3	Insulin-like growth factor binding protein 3	2.37	NS	8.08	<0.001
MMP3	Matrix metalloproteinase 3	12.58	NS	24.15	<0.05
CCL2	C–C motif chemokine ligand 2	2.59	NS	3.92	<0.01
NGF	Nerve growth factor	1.73	NS	3.18	<0.01
GREM1	Gremlin 1, DAN family BMP antagonist	0.97	NS	3.14	<0.05
SERPINE1	Serpin family E member 1	2.16	NS	3.12	<0.01
CTGF	Connective tissue growth factor	1.63	NS	2.49	<0.01
PPARδ	Peroxisome proliferator activated receptor delta	1.87	NS	2.26	<0.05
MMP2	Matrix metalloproteinase 2	1.84	NS	2.23	<0.05

*Kruskal–Wallis test with Dunn’s correction for multiple comparisons. L.D., low detection; B.D., below detection; N/A, not applicable; NS, not significant.*

**FIGURE 3:**
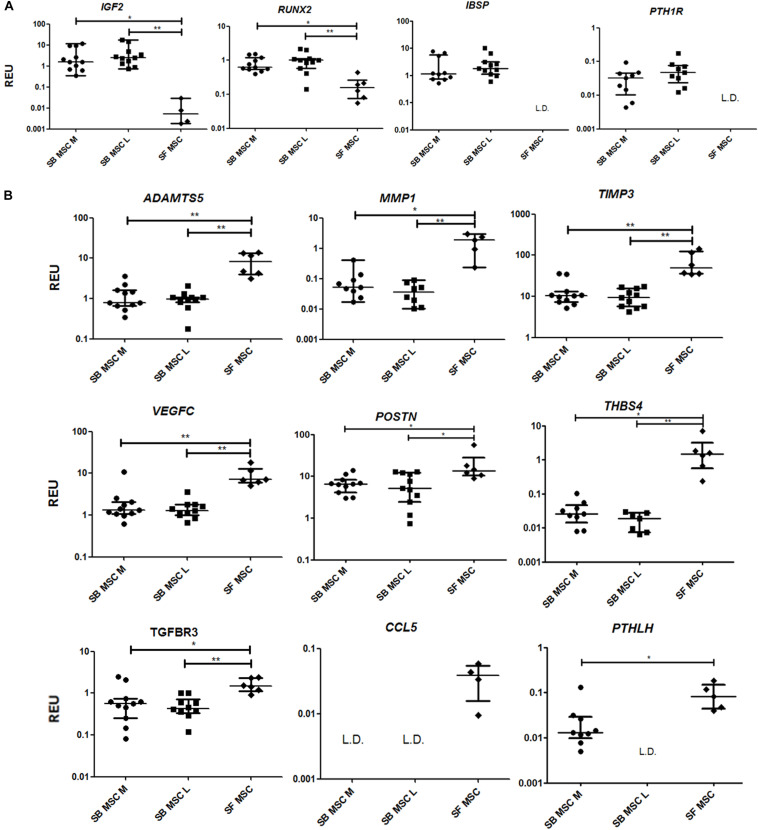
Gene expression analysis of culture-expanded medial andlateral subchondral bone (SB) multipotent stromal cells (MSCs) (SB MSC M and SB MSC L, respectively) and synovial fluid (SF) MSCs pointing toward MMP and other pathway activation in SF MSCs. **(A)** Genes expressed significantly higher in SB MSCs than SF MSCs. **(B)** Differentially expressed genes with a significant higher expression in SF MSCs than in SB MSCs. *p < 0.05; **p < 0.01; ***p < 0.0001. Horizontal bars show medians, and error bars represent interquartile range (IQR). REU, relative expression units [relative to housekeeping hypoxanthine phosphoribosyltransferase (HPRT)]. L.D. indicates transcripts which were rarely expressed (detected in <50% samples).

To summarize, compared with both medial and lateral SB MSCs, SF MSCs expressed lower levels of osteogenesis-related genes, consistent with our original hypothesis that SF MSCs were less osteogenically committed (McGonagle et al., 2017) (Figure 3A). The expression of bone-anabolic insulin growth factor 2 (IGF2) (Kang et al., 2012), and the osteogenic transcription factor (TF) Runx2, was significantly lower in SF MSCs compared with SB MSCs using both unpaired and paired tests, and the expression IBSP (encoding bone sialoprotein) displayed low detection frequency in SF MSCs, precluding its full statistical analysis using paired tests but indicating its very low expression levels in SF MSCs (Figure 3A). Expression of parathyroid hormone 1 receptor (PTH1R) was also below detection in more than 50% of SF MSCs. PTH1R regulates cartilage hypertrophy and bone turnover (Santa Maria et al., 2016) and is indirectly implicated in promoting osteoclastogenesis, along with IGF2 and prostaglandin-endoperoxide synthase 2 (PTGS2) (Benisch et al., 2012).

Compared with SB MSCs, SF MSCs expressed similar levels of classical chondrogenesis-related TF SOX9 (Supplementary Table 2) but higher levels of aggrecan (ACAN) (significant in medial SB MSCs). Unexpectedly, SF MSCs expressed significantly higher levels of genes involved in cartilage catabolism and extracellular matrix turnover (Figure 3B). The expression of matrix metalloproteinases 9 (MMP9) and 1 (MMP1), and tissue inhibitor of metalloproteinase-3 (TIMP3) and transforming growth factor receptor 3 (TGFBR3) were significantly higher in SF MSCs. Interestingly, high-level expression of extracellular matrix-degrading enzymes and their inhibitors has been previously described as a distinctive feature of OA migratory CPCs (Koelling et al., 2009). Other interesting genes expressed higher in SF MSCs were thrombospondin 4 (THBS4), serpin family E member 1 (SERPINE1), and vascular endothelial growth factor C (VEGFC), among others (Figure 3B), all previously described as involved in cartilage formation, cartilage metabolism, or cartilage changes in OA (Hissnauer et al., 2010; Seol et al., 2012; Ludin et al., 2013).

### Changes in Synovial Fluid Multipotent Stromal Cell Numbers and Colony Characteristics Following Knee Joint Distraction

**FIGURE 4:**
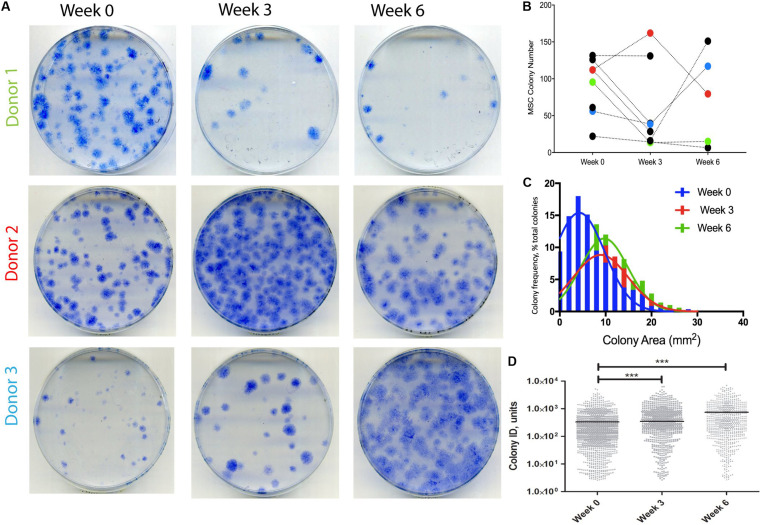
Synovial fluid (SF) multipotent stromal cell (MSC) colony numbers and characteristics following knee joint distraction (KJD). **(A)** Colonies formed by MSCs at weeks 0, 3, and 6 from three patients. **(B)** Quantification of colony numbers (total numbers); each color represents individual patient. **(C)** Colony area frequency distribution showing a shift toward larger colonies following KJD. **(D)** Quantification of colonies integrated density using Image J (1,432, 833, and 744 total colonies at weeks 0, 3, and 6, respectively). ***p < 0.0001; horizontal lines represent medians.

A different cohort of nine OA patients was next analyzed to detect any changes in SF MSCs following KJD. CFU-F assays were first performed to quantify MSCs in donor-matched SF samples before, during, and after KJD (at weeks 0, 3, and 6, respectively). Out of nine patients studied for CFU-F, three had all three time-points available (Figure 4A). No particular trend was observed in relation to colony numbers following KJD (Figure 4B). The colony areas and IDs were next quantified to assess any potential changes in MSC proliferation. Week 0 colonies were more homogenously distributed with smaller colonies compared with week 3 and 6 colonies (Figure 4C). Colony IDs, which are an integrated measure of colony area and density, were significantly higher at weeks 3 and 6 compared with week 0 (*p* < 0.001) (Figure 4D). Correspondingly, the growth rates at early passage week 3 and week 6 MSCs were higher compared with week 0 MSCs (means of 1.65, 1.67, and 1.81 days/PD, respectively), but the differences failed to reach statistical significance. Altogether, these data indicated a slight increase in SF MSC proliferative capacity following KJD.

### Changes in Synovial Fluid Multipotent Stromal Cell Gene Expression Following Knee Joint Distraction

The baseline (week 0) gene expression of SF MSCs from the KJD cohort was next compared with that of SF MSCs from OA arthroplasty cohort. No significant differences in the cell numbers at passage 0 normalized to a milliliter of SF were found between the two cohorts, indicating similarities in MSC growth potentials (Supplementary Figure 2A). Both cohorts had similarly lower bone-related IGF2, IBSP, and RUNX2 transcript expression compared with SB MSCs from the arthroplasty cohort (Supplementary Figure 2B). Similarities in the expression of other transcripts were less obvious, and the baseline (week 0) data from the KJD cohort were next compared with week 3 and 6 data to elucidate any changes in SF MSCs toward a more chondrogenic, cartilage-anabolic, or anti-catabolic phenotype following KJD.

**FIGURE 5:**
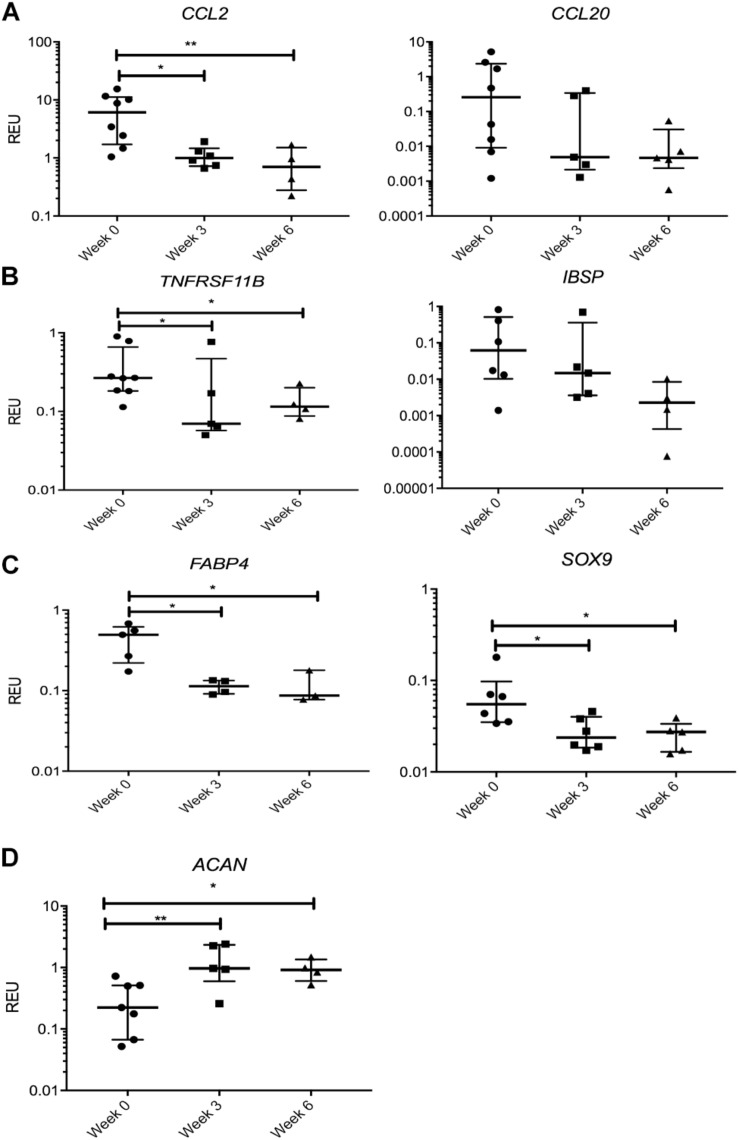
Gene expression changes in synovial fluid (SF) multipotent stromal cells (MSCs) following knee joint distraction (KJD): sustained changes. **(A)** Reduction in chemokine transcripts CCL2 and CCL20. **(B)** Reduction in bone-anabolic transcripts TNFRSF11B and IBSP. **(C)** Reduction in FABP4 and Sox9 transcripts. **(D)** Increase in ACAN transcripts. *p < 0.05; **p < 0.01; ***p < 0.0001. Horizontal bars show medians, and error bars represent interquartile range (IQR). REU, relative expression units [relative to housekeeping hypoxanthine phosphoribosyltransferase (HPRT)].

We first looked at transcripts overexpressed in SF MSCs compared with SB MSCs, and no significant changes or prominent trends were observed for IGFBP3, PPARδ, CCL5, TIMP3, and SERPINE1 (data not shown). However, a sustained nearly 10-fold reduction in the transcript levels for CCL2, encoding monocyte chemotactic protein 1 (MCP1), was observed in SF MSCs from the distracted joints (weeks 3 and 6) compared with week 0 (Figure 5A). A similar trend was observed for CCL20, encoding macrophage inflammatory protein-3 (MIP3α), but the differences failed to reach statistical significance. TNFRSF11B expression (encoding bone-anabolic osteoprotegerin) was also reduced at weeks 3 (3.8-fold) and 6 (2.5-fold) compared with week 0 (*p* < 0.05) (Figure 5B), and the expression of IBSP (encoding bone sialoprotein) was also consistently reduced albeit non-significantly. The expression of FABP4 (fatty acidic protein 4) or SOX9 (the master regulator of MSC chondrogenesis) was also reduced (Figure 5C); however, the expression of ACAN (core protein for a large cartilage proteoglycan essential for cartilage extracellular matrix maintenance) (Roughley and Mort, 2014) was, in contrast, significantly increased (Figure 5D).

Many transcripts displayed a trend for a decline following KJD but failed to reach significance, including MMP1, parathyroid hormone-like hormone (PTHLH), connective tissue growth factor (CTGF), THBS4, a disintegrin and metalloproteinase with thrombospondin motifs 5 (ADAMTS5), MMP9, and IL1b (interleukin 1 beta). Altogether, these data indicated a trend for a sustained reduction in cartilage-catabolic transcripts following KJD, with some evidence for increased ACAN expression (4.2-fold, *p* < 0.01 after 3 weeks) and (2.9-fold, *p* < 0.05 after 6 weeks) of KJD compared with its baseline levels (Figure 5D).

**FIGURE 6:**
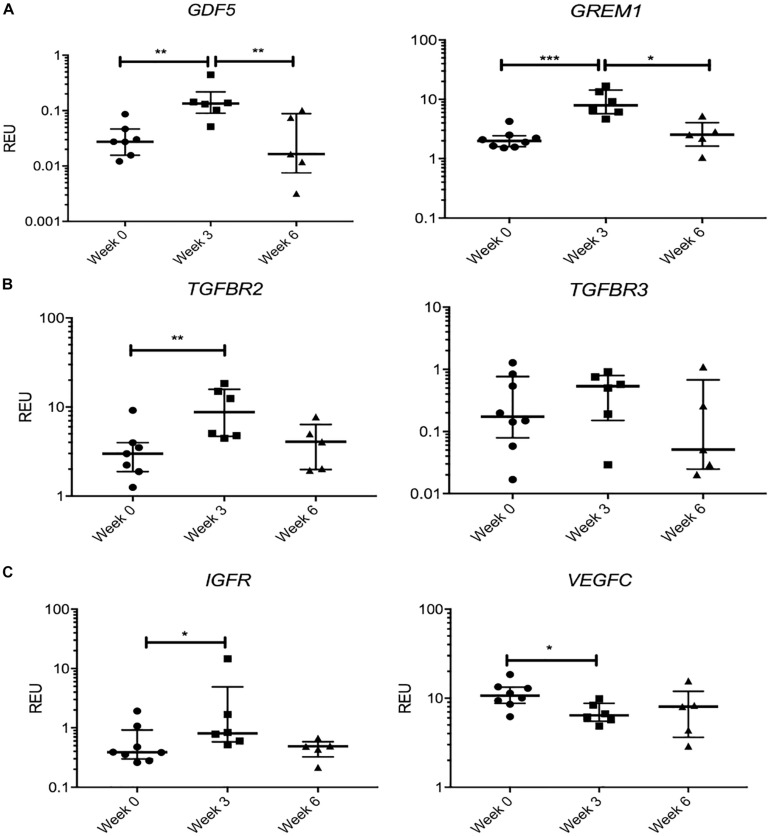
Gene expression changes in synovial fluid (SF) multipotent stromal cells (MSCs) following knee joint distraction (KJD): temporary changes. **(A)** Increase at week 3 and reduction at week 6 in progenitor-marker transcripts GDF5 and GREM1. **(B)** Similar trend in anabolic growth factor transcripts TGFBR2 and TGFBR3. **(C)** Increase in insulin growth factor receptor (IGFR) and reduction in vascular endothelial growth factor C (VEGFC) transcripts at week 3. *p < 0.05; **p < 0.01; ***p < 0.0001. Horizontal bars show medians, and error bars represent interquartile range (IQR). REU, relative expression units [relative to housekeeping hypoxanthine phosphoribosyltransferase (HPRT)].

We next tested the hypothesis that KJD may rapidly activate joint repair pathways that would regress by 6 weeks when MSC joint repair mechanisms should be well under way (Baboolal et al., 2016). Therefore, we separately considered a group of transcripts that showed a significant change in their expression levels at week 3 followed by “return to baseline” at week 6 (Figure 6). Remarkably, this group included GDF5 (4.8-fold increase at week 3, *p* < 0.01), associated with chondrogenic specification in joint interzone and synovium (Kouroupis et al., 2019) and cartilage-resident progenitors (Kania et al., 2020), and gremlin 1 (GREM1) (3.9-fold at week 3, *p* < 0.001) described as characteristic for osteochondroreticular stem cells within the metaphysis of long bones (Worthley et al., 2015) and enriched in healthy articular cartilage where it regulates hypertrophy (Leijten et al., 2013) (Figure 6A). A similar pattern of changes was seen for TGFBR2 and TGFBR3 (Figure 6B) and IGFR (Figure 6C), the receptors for cartilage-anabolic growth factors and involved in pathogenesis of OA (Goldring, 2000). VEGFC, a well-known pro-angiogenic factor, showed a small decrease (1.6-fold, *p* < 0.05) at week 3 and returned to baseline after 6 weeks (Figure 6C). The VEGF family have been associated with OA progression in all tissues in the joint (Hamilton et al., 2016) and found strongly expressed in hyperplasic osteoarthritic synovium (Paavonen et al., 2002).

Overall, this pattern of gene expression indicated an early upregulation of genes associated with chondrogenic lineage specification and responsiveness to cartilage-anabolic growth factors, which may explain a more sustained increase in ACAN expression. At the end of KJD, there was also reduction in a pro-inflammatory CCL2/MCP-1 gene expression in SF MSCs as well as downregulation of typical bone- and fat-lineage genes.

## Discussion

Novel therapeutic interventions based on the modulation of local biomechanical or biological environments within the OA-affected joint have recently emerged as potential joint-sparing alternatives to joint replacement surgery (Sánchez et al., 2012; Jansen et al., 2018; Goh et al., 2019; Jansen et al., 2019; Takahashi et al., 2019). Harnessing endogenous joint repair mechanisms following biomechanical correction of OA joints following KJD appears to hold particular promise (Mastbergen et al., 2013; McGonagle et al., 2017). While their mechanisms of action remain to be fully elucidated, endogenous SB and SF MSCs have been suggested as potential contributors to structural improvements and cartilage regeneration following these treatments (Chen et al., 2015; Baboolal et al., 2016; Sánchez et al., 2016). In this study, we investigated gene expression profiles of SF and SB MSCs in advanced knee OA and explored whether KJD, a successful treatment for advanced OA, leads to any favorable cartilage-anabolic changes in SF MSCs.

At the beginning, we compared gene expression profiles of SF MSCs and SB MSCs with articular chondrocytes enzymatically extracted from the same joints. Our results showed clear clustering of chondrocytes away from both types of MSCs indicating toward less-differentiated nature of both types of MSCs. Recent studies have indicated the existence, within the OA cartilage, of highly proliferative and migrating CPCs (Koelling et al., 2009; Fellows et al., 2017; Carluccio et al., 2020), which could in theory, be shed into the SF and contribute to increased levels of matrix-turnover molecules in SF MSCs. Chondrocyte cultures, which were derived from the full-depth OA cartilage in the present study, could also contain the progeny of these migrating CPCs. Our data on chondrocyte cultures showed their higher-level expression of typical cartilage-specific molecules, compared with MSCs, as well as some evidence of chondrocyte inflammation, based on increased expression of pro-inflammatory molecules lipocalin-2 (LCN2) (Choi and Chun, 2017), NOS2 (Ahmad et al., 2020), CCL20 (Alaaeddine et al., 2015), and C–C motif chemokine receptor 7 (CCR7). These data indicated clear differences in gene expression profiles between cultured full-depth cartilage-resident cells and MSCs from the same late-OA joints and pointed toward cartilage degradation and inflammation as potential contributing factors to MSC gene expression signatures in late-stage OA joints.

In agreement with Djouad et al. (2005) for healthy bone marrow MSCs and SF MSCs, SB MSCs and SF MSCs formed separate sub-clusters within the MSC cluster in our study. This could indicate different tissue origins of these MSCs (Sekiya et al., 2012; McGonagle et al., 2017), but this could also be a reflection of different biomechanical environments that these MSCs reside in. We have previously shown that SB MSCs from medial (more loaded) femoral condyles had a gene expression profile enriched for bone-anabolic genes compared with MSCs from lateral (less loaded) condyles, implicating joint biomechanics as a potential main driver of SB MSC commitment to osteogenesis and sclerotic plate formation in late-OA joints (Sanjurjo-Rodriguez et al., 2019). In the present study, SF MSCs under-expressed many of these osteogenic and hypertrophy-related genes compared with SB MSCs (IGF2, RUNX2, IBSP, and PTH1R) supporting SF MSC role in endogenous cartilage repair *in vivo* in experimental OA (Wiegant et al., 2015) and arguing for better suitability of SF MSCs for cartilage regeneration in OA in humans (Baboolal et al., 2016).

The lack of a clear pro-chondrogenic or cartilage anabolic phenotype of OA SF MSCs could at least in part be related to pro-inflammatory mediators present in late-OA SF (Monibi et al., 2016). Indeed, OA SF MSCs expressed higher levels of many genes related to cartilage catabolism and extracellular matrix turnover (including ADAMTS5, MMP1, and TIMP3) compared with SB MSCs. This is similar to CPCs, which express high levels of these molecules, possibly to facilitate their tissue egress and remodeling of damaged extracellular matrix (Koelling et al., 2009). Such a unique gene expression profile may also reflect a strong effect of cartilage breakdown and joint inflammation on SF MSCs (Leijs et al., 2012; Neybecker et al., 2018). Regardless of the cause, this gene expression profile might be of relevance to remodeling the damaged cartilage matrix and enabling endogenous repair by SF MSCs.

Higher numbers, but reduced functionalities of MSCs in OA SF compared with healthy donors, have been reported in many previous studies (Jones et al., 2008; Sekiya et al., 2012; Kim et al., 2015), but the precise mechanisms behind this unexpected increase remain unknown. The molecular and metabolic milieu of OA SF may encourage resident MSC self-renewal (Jones et al., 2008; Harris et al., 2013), but it may also induce the egress of CPCs and SB MSCs from their natural niches (Sánchez et al., 2016; De Luca et al., 2019) or reduce their homing and attachment to damaged cartilage and other joint tissues (Endres et al., 2007; Baboolal et al., 2016; De Luca et al., 2019). Another promising joint-preserving regenerative treatment for severe OA, a combination of intra-articular and intra-osseous infiltrations of autologous platelet-rich plasma (PRP), has been shown to reduce SF MSC numbers measured by flow cytometry (Muiños-López et al., 2016; Sánchez et al., 2016), presumably by attraction of MSCs to the exposed osseous surface. In our cohort of KJD patients, SF MSC numbers measured by CFU-F assay did not increase or fall following the treatment, although higher colony densities during treatment pointed toward their slightly increased proliferative capacity that may in part be driven by KJD-associated changes in SF FGF2 concentration (Coutu et al., 2011; Watt et al., 2020). We acknowledge that aspirated SF MSC numbers could not be volumetrically counted, as the volumes of fluid taken from the patients could not be precisely controlled and depended on patient’s clinical status; therefore, the data were presented as total aspirated MSCs. Nevertheless, SF MSC responses to KJD appeared to be different to those reported following PRP infiltrations.

Our analysis of SF MSC transcriptome half-way through (week 3) and in the end of KJD (week 6) provided interesting insights into the dynamics of SF MSC responses in relation to previously reported SF cytokine changes (Watt et al., 2020). In a separate cohort of 20 KJD patients, mild but sustained increases in SF levels of FGF2 and TGFb1 have been recently documented, which were particularly prominent in patients who responded better to their treatment (Watt et al., 2020). Our data showed an increase in TGFβ receptor 2 and 3 expression in SF MSCs early in KJD, which may represent an enrichment in TGFβ-responsive MSCs during early stages of treatment. Interestingly, this coincided with higher-level GREM1 and GDF5 transcripts in week 3 MSCs, the molecules associated with chondrogenesis and healthy cartilage homeostasis (Leijten et al., 2013; Worthley et al., 2015; Kouroupis et al., 2019; Kania et al., 2020). A sustained increase in ACAN expression in SF MSCs following KJD suggests some degree of their chondrogenic commitment and may be explained by the activity of these GREM1- and GDF5-expressing cells.

It is difficult to directly compare the gene expression of SF MSCs following KJD with our results obtained for their comparison with SB MSCs, as different patient cohorts were used. Some key similarities in SF MSCs from both cohorts were however found, particularly in relation to their reduced levels of osteogenic transcripts IGF2, IBSP, and RUNX2, compared with SB MSCs, as well as similar growth potentials. However, it was noted that several genes higher expressed in the OA SF MSCs and assumed to be upregulated in response to pro-inflammatory SF mediators (ADAMTS5, IL1b, MMP9, or MMP1) in our OA arthroscopy cohort did not show a significant change following KJD, but they tended to decrease. Interestingly, SF MSC expression of pro-inflammatory chemokines (CCL2/MCP1 and CCL20/MIP1α) was reduced. MCP1 is associated with synovial inflammation (Wang et al., 2013) and implicated in joint pain (Miotla Zarebska et al., 2017), as well as an inhibition of SF MSC chondrogenesis (Harris et al., 2013). In contrast to an animal model studies where a significant effect of KJD on joint inflammation was found (Chen et al., 2015; Wiegant et al., 2015), early results on the effects of KJD on SF pro-inflammatory mediators in OA patients remain puzzling. For example, pro-inflammatory IL-6 and MCP1 SF levels were found increased following KJD in Watt et al. (2020) study, although IL-6 was negatively correlated with pro-chondrogenic TGFb1, as would be expected (Wiegertjes et al., 2019). Larger studies investigating more SF analytes and SF MSC transcripts using the same cohort of patients (validation cohort), ideally at more time-points, during the course of distraction and comparing responders and non-responders, would be needed to shed more light on the effects of joint off-loading on SF cellular and molecular responses. Changes in SF MSC secretome and their immunomodulation, chondrogenic and chondroprotective potencies using appropriate assays (Cassano et al., 2018; Amemiya et al., 2020; Watanabe et al., 2020) would be needed to confirm the functional significance of the observed transcript changes. In addition to MSCs, the investigations of SF immune cells, and particularly macrophage subsets in terms of their pro- and anti-inflammatory polarization states (Fahy et al., 2014; Daghestani et al., 2015), would be necessary.

This study is limited by small numbers of samples tested and by the analysis of SB and SF MSC expression in culture-expanded MSCs, which is known to influence gene expression (Churchman et al., 2012; Sanjurjo-Rodriguez et al., 2019). We have previously demonstrated the feasibility of analyzing gene expression in uncultured SB MSCs from knee OA patients where differentially expressed genes between lateral and medial femoral condyles were discovered using cultured MSCs and subsequently confirmed using CD271-selected uncultured MSCs (Sanjurjo-Rodriguez et al., 2019). However, the isolation of uncultured SF MSCs, which are significantly rarer (Jones et al., 2004, 2008; Morito et al., 2008; Altaie et al., 2018), remains a challenge. New methodologies involving single-cell quantitative PCR or RNA sequencing, as utilized for other types of rare stem cells (Ayyaz et al., 2019; Tricoire et al., 2019; Zhou et al., 2019), provide a future way forward. Our chondrocyte cultures were derived from full-depth OA cartilage and grown in culture media different from MSC-expansion media; this could have partially contributed to differences in their gene expression compared with MSCs. Similarly, single-cell RNA sequencing (Ji et al., 2019) or mass cytometry (Grandi et al., 2020) on cartilage-resident cells including uncultured CPCs and comparing their profiles with single MSCs would provide future valuable insights into different progenitor and stem cell types present in OA-affected human joints. If cultured, the same early passage cells should be used for transcript analysis and functional assays.

Apart from SF MSCs, joint off-loading following KJD is likely to have an impact on SB MSCs, but bone biopsy was considered too invasive and therefore deemed unethical. The assessment of SB microstructural changes using CT scans (Intema et al., 2011) and correlating them with SB MSC changes and cartilage regeneration (Hyodo et al., 2019) would be valuable for future evaluation of the roles of SB MSCs in osteochondral structural improvements and cartilage metabolism (Westacott et al., 1997) following KJD. Finally, primary biomechanical effects of joint off-loading are also possible, for example, SF viscosity changes, hyaluronan content, or cartilage swelling, which are potential drivers for SF attachment to cartilage occurred within the first week following off-loading (Baboolal et al., 2016) and were therefore missed in the present study protocol. Future studies should also include investigations of synovial MSC subpopulations and comparing them, through high-throughput single cell-based analysis, to SF MSCs. Up to now, there exist multiple studies presenting different phenotypes and topographies of MSCs in human synovium (Sivasubramaniyan et al., 2012; O’Brien et al., 2017; Mizuno et al., 2018; Affan et al., 2019) or rodent synovium (Roelofs et al., 2017); and their relationships with synovial fibroblasts (Croft et al., 2019), which may be pro-inflammatory, remain unclear. In the future, it would be interesting to establish which subset of synovial MSCs may be preferentially shed into the fluid, using recently developed *in vitro* methodologies (Kohno et al., 2018). Endogenous manipulations of the osteoarthritic synovium toward inflammation inhibition and increased MSC shedding into the fluid could be the next step toward KJD efficacy augmentation.

Our findings have broad implications for the development of novel joint-sparing regenerative strategies as they highlight the complexity of different joint-resident MSC niches, MSC responses to the disease itself, and dynamic changes favoring tissue repair following biomechanical correction by joint off-loading. Many clinical investigations employ MSC intra-articular injections as a sole therapy based on their tissue repair and immunomodulatory activities *in vitro* (Chahal et al., 2019; Matas et al., 2019), but long-term clinical benefits are not always guaranteed (Shariatzadeh et al., 2019). Our data highlight the importance of considering complex cellular and biomechanical environments into which these cells are delivered for the development of more effective joint-sparing treatments for OA.

## Data Availability Statement

The analyzed datasets for this study can be found in the University of Leeds data depository on https://doi.org/10.5518/837.

## Ethics Statement

The studies involving human participants were reviewed and approved by Yorkshire & The Humber – South Yorkshire Research Ethics Committee (14/YH/0087), UK; University Medical Center Utrecht (#15-160/D; NL51539.041.15), Netherlands. The patients/participants provided their written informed consent to participate in this study.

## Author Contributions

EJ, DM, FL, SM, TB, and CS-R performed conception and design. HP, CS-R, AA, TW, SM, and TB acquired data. CS-R, AA, TB, and EJ performed the data analysis. CS-R, AA, EJ, TB, SM, FL, TW, and DM carried out data interpretation. CS-R, AA, and EJ contributed to manuscript preparation. All authors performed manuscript review and editing for important intellectual content.

## Conflict of Interest

The authors declare that the research was conducted in the absence of any commercial or financial relationships that could be construed as a potential conflict of interest.

## Supplementary Material

Supplementary Figure 1 Tissue processing **(A)** and the immunohistochemical staining of an example osteochondral specimen **(B)** showing an absence of intact cartilage superficial layer and the presence of CD271 + MSCs in marrow cavities (dotted arrows) and in bone-lining locations (black arrows). Dotted line represents an approximate position of slicing-off the cartilage using a scalpel. The method for processing and immunohistochemical staining of OA knee osteochondral specimens can be found in “Materials and Methods” and “Supplementary Material”.

Supplementary Figure 2 Comparison between SF MSCs from OA arthroplasty (OA/A) cohort and SF MSCs from KJD cohort at baseline (week 0). Similar growth potentials were observed **(A)** (lines represent medians, each symbol represents an individual donor). Gene expression heatmap of SF MSCs from both cohorts: OA/A (*n* = 6), KJD (*n* = 8), and SB MSCs from OA/A cohort (*n* = 11) used as comparator **(B)**. Heatmap is generated using Graphpad Prism version 8.4.3. Log2 transformation and data filtering (filter = 67% present) were performed on the data and color coding represents medians. Osteogenic genes differentially expressed between SB MSCs and both groups of SF MSCs are shown in yellow brackets.

Supplementary Table 1 Taqman probes used to assess the gene expression.

Supplementary Table 2 Genes not differentially expressed between SF and SB MSCs.
