# Supplementary material for: Gene Expression Signatures of Synovial Fluid Multipotent Stromal Cells in Advanced Knee Osteoarthritis and Following Knee Joint Distraction
Source: Front Bioeng Biotechnol. 2020 Oct 14;8:579751. doi: 10.3389/fbioe.2020.579751 (PMC7591809; doi:10.3389/fbioe.2020.579751)
Supplement: Supplementary Figure 1 — Tissue processing (A) and the immunohistochemical staining of an example osteochondral specimen (B) showing an absence of intact cartilage superficial layer and the presence of CD271 + MSCs in marrow cavities (dotted arrows) and in bone-lining locations (black arrows). Dotted line represents an approximate position of slicing-off the cartilage using a scalpel. The method for processing and immunohistochemical staining of OA knee osteochondral specimens can be found in “Materials and Methods” and “Supplementary Material”. [file Data_Sheet_1.docx]

Supplementary Material

# Supplementary Tables

## Supplementary Table 1

**Supplementary Table 1.** The Taqman probes used to assess the gene expression

| **Gene symbol** | **Gene name** | **Assay number** |
| --- | --- | --- |
| ACAN | aggrecan | HS00153936-m1 |
| ADAMTS4 | a disintegrin and metalloproteinase with thrombospondin motifs 4 | HS00192708-m1 |
| ADAMTS5 | a disintegrin and metalloproteinase with thrombospondin motifs 5 | HS01095524-m1 |
| ANKH | human homolog of the murine progressive ankylosis gene | HS01064613-m1 |
| ARNTL | aryl hydrocarbon receptor nuclear translocator-like protein 1 | HS00154147-m1 |
| ASPN | asporin | HS01558901-m1 |
| BCL2 | BCL2, apoptosis regulator | HS00608023-m1 |
| BGLAP | Bone Gamma-Carboxyglutamate Protein | HS01587814-g1 |
| BMPR1B | Bone Morphogenetic Protein Receptor Type 1B | HS01010965-m1 |
| CCL19 | C-C Motif Chemokine Ligand 19 | HS00171149-m1 |
| CCL2 | C-C Motif Chemokine Ligand 2 | HS00234140-m1 |
| CCL20 | C-C Motif Chemokine Ligand 20 | HS01011368-m1 |
| CCL5 | C-C Motif Chemokine Ligand 5 | HS00982282-m1 |
| CCR1 | C-C Motif Chemokine Receptor 1 | HS00174298-m1 |
| CCR10 | C-C Motif Chemokine Receptor 10 | HS00706455-s1 |
| CCR2 | C-C Motif Chemokine Receptor 2 | HS00356601-m1 |
| CCR3 | C-C Motif Chemokine Receptor 3 | HS99999027-s1 |
| CCR5 | C-C motif chemokine receptor 5 | HS00152917-m1 |
| CCR6 | C-C Motif Chemokine Receptor 6 | HS00171121-m1 |
| CCR7 | C-C Motif Chemokine Receptor 7 | HS04398702-m1 |
| COL10A1 | Collagen Type X Alpha 1 Chain | HS00166657-m1 |
| COL1A1 | Collagen Type I Alpha 1 Chain | HS01076777-m1 |
| COL1A2 | Collagen Type I Alpha 2 Chain | HS01028971-m1 |
| COL2A1 | Collagen Type II Alpha 1 Chain | HS00264051-m1 |
| COMP | Cartilage Oligomeric Matrix Protein | HS00164359-m1 |
| CTGF | Connective Tissue Growth Factor | HS00170014-m1 |
| CXCL12 | C-X-C Motif Chemokine Ligand 12 | HS00171022-m1 |
| CXCR1 | C-X-C Motif Chemokine Receptor 1 | HS01921207-m1 |
| CXCR4 | C-X-C Motif Chemokine Receptor 4 | HS00607978_S1 |
| CYR61 | Cysteine Rich Angiogenic Inducer 61 | HS00155479-m1 |
| DDR2 | iscoidin Domain Receptor Tyrosine Kinase 2 | HS01025956-m1 |
| DIO2 | Iodothyronine Deiodinase 2 | HS00255341-m1 |
| DIRAS2 | DIRAS Family GTPase 2 | HS01107862-m1 |
| FABP4 | Fatty Acid Binding Protein 4 | HS00609791-m1 |
| GDF5 | Growth Differentiation Factor 5 | HS00167060-m1 |
| GDF6 | Growth Differentiation Factor 6 | HS01377663-m1 |
| GREM1 | Gremlin 1, DAN Family BMP Antagonist | HS00171951-m1 |
| HGF | Hepatocyte Growth Factor | HS00300159-m1 |
| **HPRT1** | Hypoxanthine Phosphoribosyltransferase 1 | HS99999909-m1 |
| IBSP | integrin binding sialoprotein | HS00173720-m1 |
| IGF1 | Insulin Like Growth Factor 1 | HS03986524-m1 |
| IGF1R | Insulin Like Growth Factor 1 Receptor | HS00609566-m1 |
| IGF2 | Insulin Like Growth Factor 2 | HS00171254-m1 |
| IGFBP3 | Insulin Like Growth Factor Binding Protein 3 | HS00426289-m1 |
| IL10 | Interleukin 10 | HS00961622-m1 |
| IL1B | Interleukin 1 Beta | HS01555413-m1 |
| IL6 | Interleukin 6 | HS00174131-m1 |
| LCN2 | Lipocalin 2 | HS01008571-m1 |
| LEPR | Leptin Receptor | HS00174492-m1 |
| MMP1 | Matrix Metalloproteinase 1 | HS00899658-m1 |
| MMP13 | Matrix Metalloproteinase 13 | HS00942589-m1 |
| MMP14 | Matrix Metalloproteinase 14 | HS00237119-m1 |
| MMP2 | Matrix Metalloproteinase 2 | HS01548728-m1 |
| MMP3 | Matrix Metalloproteinase 3 | HS00968308-m1 |
| MMP9 | Matrix Metalloproteinase 9 | HS00957562-m1 |
| NGF | Nerve Growth Factor | HS00171458-m1 |
| NGFR | Nerve Growth Factor Receptor | HS00182120-m1 |
| NOS2 | Nitric Oxide Synthase 2 | HS01075529-m1 |
| NOTCH1 | Notch 1 | HS01062014_m1 |
| POSTN | Periostin | HS01566750-m1 |
| PPARd | Peroxisome Proliferator Activated Receptor Delta | HS00602622-m1 |
| PPARg | Peroxisome Proliferator Activated Receptor Gamma | HS01115513-M1 |
| PSIP1 | PC4 And SFRS1 Interacting Protein 1 | HS01045714-g1 |
| PTGS2 | Prostaglandin-Endoperoxide Synthase 2 | HS00153133-m1 |
| PTH1R | Parathyroid Hormone 1 Receptor | HS00896824-m1 |
| PTHLH | Parathyroid Hormone Like Hormone | HS00174969-m1 |
| ROR2 | Receptor Tyrosine Kinase Like Orphan Receptor 2 | HS00171695-m1 |
| RUNX2 | Runt Related Transcription Factor 2 | HS00234692-m1 |
| S1PR1 | Sphingosine-1-Phosphate Receptor 1 | HS00173499-m1 |
| SERPINE1 | Serpin Family E Member 1 | HS00167155-m1 |
| SFRP1 | Secreted Frizzled Related Protein 1 | HS00610060-m1 |
| SFRP4 | Secreted Frizzled Related Protein 4 | HS00180066-m1 |
| SOX9 | SRY-Box 9 | HS00165814-m1 |
| SP7 | Sp7 Transcription Factor | HS00541729-m1 |
| SPARC | Secreted Protein Acidic And Cysteine Rich | HS00277762-m1 |
| SPHK1 | Sphingosine Kinase 1 | HS00184211-m1 |
| SPP1 | Secreted Phosphoprotein 1 | HS00959010-m1 |
| STMN2 | Stathmin 2 | HS00975800-m1 |
| TGFB1 | transforming growth factor beta 1 | HS00998133-m1 |
| TGFB2 | transforming growth factor beta 2 | HS00234244-m1 |
| TGFB3 | Transforming Growth Factor Beta 3 | HS01085997-m1 |
| TGFBR1 | Transforming Growth Factor Beta Receptor 1 | HS00610319-m1 |
| TGFBR2 | transforming growth factor beta receptor 2 | HS00559661-m1 |
| TGFBR3 | Transforming Growth Factor Beta Receptor 3 | HS00234257-m1 |
| THBS4 | Thrombospondin 4 | HS00170261-m1 |
| TIMP1 | Tissue inhibitor of metalloproteinase- 1 | HS00171558-m1 |
| TIMP2 | Tissue inhibitor of metalloproteinase- 2 | HS01091319-m1 |
| TIMP3 | Tissue inhibitor of metalloproteinase- 3 | HS00927214-m1 |
| TNFa | Tumor Necrosis Factor | HS99999043-m1 |
| TNFRSF11B | TNF Receptor Superfamily Member 11b | HS00900360-m1 |
| TNFSF11 | TNF Superfamily Member 11 | HS01092186-m1 |
| VEGFA | Vascular Endothelial Growth Factor A | HS00900058-m1 |
| VEGFC | Vascular Endothelial Growth Factor C | HS01099206-m1 |
| WISP1 | WNT1 Inducible Signaling Pathway Protein 1 | HS04234730-m1 |
| WNT10b | Wnt Family Member 10B | HS00559664-m1 |

## Supplementary Table 2

**Supplementary Table 2.** Genes not differentially expressed between SF and SB MSCs.

| **Similar expression in SF and SB MSCs** | |
| --- | --- |
| **Gene symbol** | **Gene name** |
| ARNTL | Aryl hydrocarbon receptor nuclear translocator-like protein 1 |
| ADAMTS4 | A disintegrin and metalloproteinase with thrombospondin motifs 4 |
| ANKH | Human homolog of the murine progressive ankylosis gene |
| BCL2 | BCL2, apoptosis regulator |
| COL1A1 | Collagen Type I Alpha 1 Chain |
| COL1A2 | Collagen Type I Alpha 2 Chain |
| CYR61 | Cysteine Rich Angiogenic Inducer 61 |
| DIO2 | Iodothyronine Deiodinase 2 |
| FABP4 | Fatty Acid Binding Protein 4 |
| GDF5 | Growth Differentiation Factor 5 |
| GDF6 | Growth Differentiation Factor 6 |
| HGF | Hepatocyte Growth Factor |
| IGF1R | Insulin Like Growth Factor 1 Receptor |
| MMP13 | Matrix Metalloproteinase 13 |
| MMP14 | Matrix Metalloproteinase 14 |
| Notch1 | Notch 1 |
| PPARG | Peroxisome Proliferator Activated Receptor Gamma |
| PSIP1 | PC4 And SFRS1 Interacting Protein 1 |
| ROR2 | Receptor Tyrosine Kinase Like Orphan Receptor 2 |
| S1PR1 | Sphingosine-1-Phosphate Receptor 1 |
| SFRP1 | Secreted Frizzled Related Protein 1 |
| SFRP4 | Secreted Frizzled Related Protein 4 |
| SOX9 | SRY-Box 9 |
| SPARC | Secreted Protein Acidic And Cysteine Rich |
| SPHK1 | Sphingosine Kinase 1 |
| SPP1 | Secreted Phosphoprotein 1 |
| TGFB1 | Transforming growth factor beta 1 |
| TGFB2 | Transforming growth factor beta 2 |
| TGFB3 | Transforming Growth Factor Beta 3 |
| TGFBR1 | Transforming Growth Factor Beta Receptor 1 |
| TIMP1 | Tissue inhibitor of metalloproteinase- 1 |
| TNFRSF11B | TNF Receptor Superfamily Member 11b |
| TNFSF11 | TNF Superfamily Member 11 |
| VEGFA | Vascular Endothelial Growth Factor A |
| WISP1 | WNT1 Inducible Signaling Pathway Protein 1 |
| **Expression below detection in both SF and SB MSCs** | |
| **Gene symbol** | **Gene name** |
| ASPN | Asporin |
| BGLAP | Bone Gamma-Carboxyglutamate Protein |
| BMPR1B | Bone Morphogenetic Protein Receptor Type 1B |
| CCL19 | C-C Motif Chemokine Ligand 19 |
| CCL20 | C-C Motif Chemokine Ligand 20 |
| CCR10 | C-C Motif Chemokine Receptor 10 |
| CCR2 | C-C Motif Chemokine Receptor 2 |
| CCR3 | C-C Motif Chemokine Receptor 3 |
| CCR5 | C-C motif chemokine receptor 5 |
| CCR6 | C-C Motif Chemokine Receptor 6 |
| CCR7 | C-C Motif Chemokine Receptor 7 |
| COL10A1 | Collagen Type X Alpha 1 Chain |
| COL2A1 | Collagen Type II Alpha 1 Chain |
| COMP | Cartilage Oligomeric Matrix Protein |
| CXCR1 | C-X-C Motif Chemokine Receptor 1 |
| CXCR4 | C-X-C Motif Chemokine Receptor 4 |
| DIRAS2 | DIRAS Family GTPase 2 |
| IGF1 | Insulin Like Growth Factor 1 |
| IL10 | Interleukin 10 |
| IL1B | Interleukin 1 Beta |
| IL6 | Interleukin 6 |
| LCN2 | Lipocalin 2 |
| NGFR | Nerve Growth Factor Receptor |
| NOS2 | Nitric Oxide Synthase 2 |
| TIMP2 | Tissue inhibitor of metalloproteinase- 2 |
| TNFa | Tumor Necrosis Factor |
| Wnt10b | Wnt Family Member 10B |

# Supplementary Materials and methods

**2.1. Immunohistochemistry**

As described before (Sanjurjo-Rodriguez, et al. 2019), whole condyles were fixed for 1 week in 3.7% formaldehyde (Thermofisher Scientific, UK), decalcified for at least 6 months using 0.5M ethylenediaminetetraacetic acid (EDTA; Sigma, USA), re-fixed for further 2 days in formaldehyde before embedding in paraffin blocks. Immunohistochemical staining for CD271 was performed using monoclonal mouse anti-human antibody (clone ME20.4; Invitrogen, USA) at 1:200 dilution and EnVision+ Dual Link System-HRP (DAB+) (Dako, Agilent, USA). Sections were counterstained with hematoxylin and the slides scanned using Leica Aperio AT2.

# Supplementary Figures

**3.1. Supplementary Figure 1**


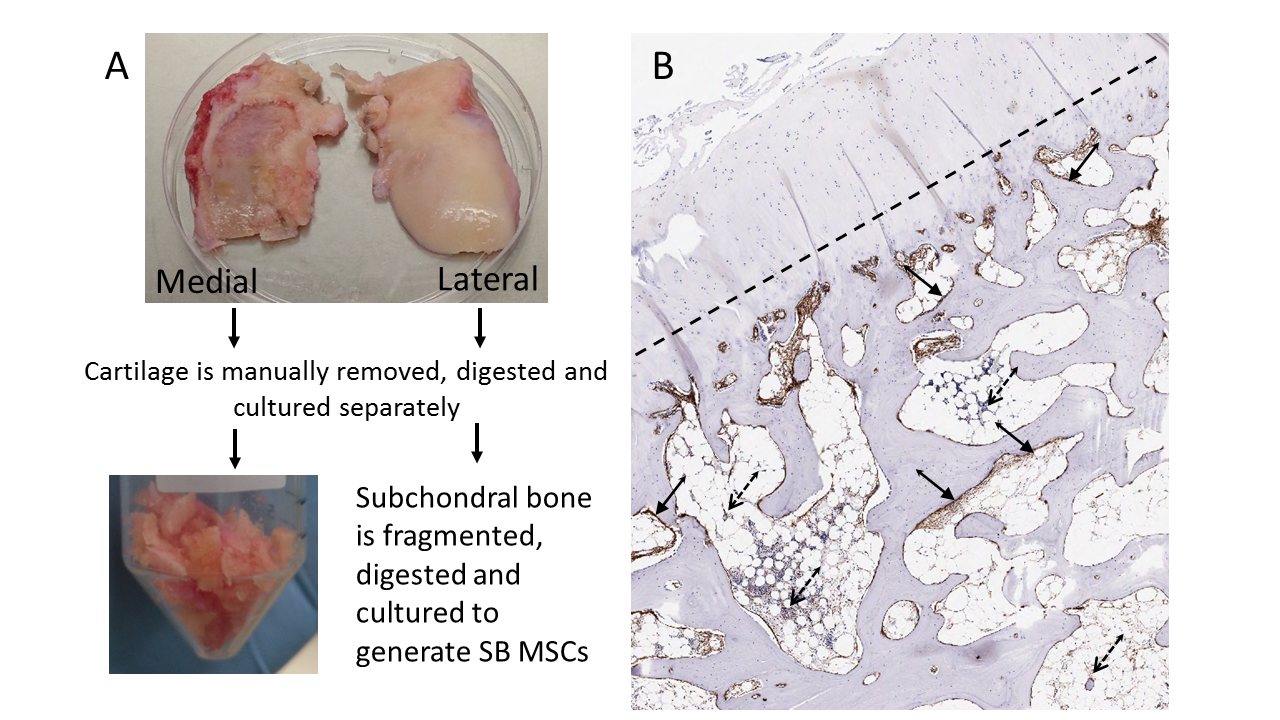


**Supplementary Figure 1:** Tissue processing (A) and the immunohistochemical staining of an example osteochondral specimen (B) showing an absence of intact cartilage superficial layer and the presence of CD271+ MSCs in marrow cavities (dotted arrows) and in bone-lining locations (black arrows). Dotted line represents an approximate position of slicing-off the cartilage using a scalpel. The method for processing and immunohistochemical staining of OA knee osteochondral specimens can be found in “Material and methods” and “Supplementary Materials and methods”.

**3.2. Supplementary Figure 2.**


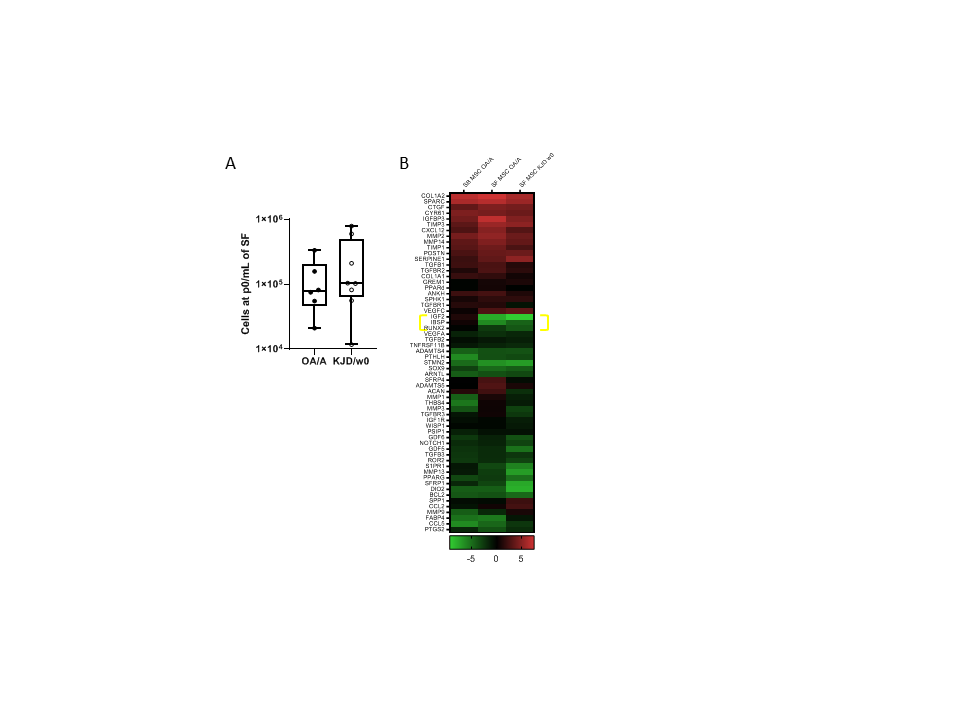


**Supplementary Figure 2:** Comparison between SF MSCs from OA arthroplasty (OA/A) cohort and SF MSCs from KJD cohort at baseline (week 0). Similar growth potentials were observed (A) (lines represent medians, each symbol represents an individual donor). Gene expression heatmap of SF MSCs from both cohorts: OA/A (n=6), KJD (n=8), and SB MSCs from OA/A cohort (n=11) used as comparator (B). Heatmap is generated using Graphpad Prism version 8.4.3. Log2 transformation and data filtering (filter = 67% present) were performed on the data and colour coding represents medians. Osteogenic genes differentially expressed between SB MSCs and both groups of SF MSCs are shown in yellow brackets.
